# Feasibility study of an AI-powered mobile app to support cutaneous leishmaniasis diagnosis in the Brazilian Amazon

**DOI:** 10.1371/journal.pntd.0014313

**Published:** 2026-05-27

**Authors:** Karina Lumy Okita, Thamires Bastos Pinheiro, Livia Oliveira-Ciabati, Breno dos Santos Alves, Pedro Luis Gil Bonett, Elinea Liborio Fragoso, Eddy Jens Rivero-Zavala, Antonio Joaquim Fernandes, Gustavo Costa Souza, Jorge Ewerton dos Santos Sales, Maria das Graças Vale Barbosa Guerra, Jorge Augusto de Oliveira Guerra, Isabelle Carvalho

**Affiliations:** 1 Health Innovation Techcenter, Hospital Israelita Albert Einstein: Sociedade Beneficente Israelita Brasileira Albert Einstein, São Paulo, São Paulo, Brasil; 2 Dermatology Department, Universidade de Mogi das Cruzes, Mogi das Cruzes, São Paulo, Brasil; 3 Program in Applied Sciences in Dermatology at the State University of Amazonas, Manaus, Amazonas, Brasil; 4 Centro Universitário Barão de Mauá, Ribeirão Preto, São Paulo, Brasil; 5 Fundação Hospitalar Alfredo da Matta, Manaus, Amazonas, Brasil; 6 Fundação de Medicina Tropical Doutor Heitor Vieira Dourado, Manaus, Amazonas, Brasil; University of Colombo Faculty of Medicine, SRI LANKA

## Abstract

**Background:**

Cutaneous leishmaniasis (CL) remains a major public health challenge, especially in Brazil’s Amazon, where environmental and economic pressures sustain transmission. Delayed diagnosis drives morbidity, stigma, and costs. Community health workers are pivotal yet under-equipped for early triage. Artificial intelligence, effective in dermatologic imaging, is underused for CL; feasible, offline clinical tools could accelerate referral and timely care. This study aimed to evaluate the feasibility of an AI-assisted tool for early CL triage in the Brazilian Amazon by: (i) developing an AI model for CL identification from clinical skin lesion images; (ii) integrating the model into an offline-optimized mobile application for resource-limited settings; and (iii) conducting initial, real-world clinical validation.

**Methods:**

Exploratory, IRB-approved feasibility study. Retrospective images from Brazilian Amazon sites informed a two-stage AI pipeline (lesion segmentation+classification) integrated into an offline mobile app. Multicenter validation in ideal- and real-world scenarios. Primary metric AUC-ROC; secondary sensitivity/specificity. One-sided tests: AUC-ROC >0.70; ideal-world sensitivity >0.75.

**Results:**

For the Classification Model, 64 images were assigned to the test set while 1,160 were used for training and validation (80:20 split), with DenseNet121 yielding the highest accuracy of 0.88. The full AI pipeline (Segmentation and Classification models) achieved an accuracy of 0.81, an F1-score of 0.80, an AUC-ROC of 0.90, and a sensitivity of 0.76. In the ideal-world analysis, sensitivity reached 0.92, the F1-score was 0.84, and specificity was 0.42.

**Conclusions:**

We demonstrate the feasibility of an offline, AI-assisted mobile tool to support triage and referral for cutaneous leishmaniasis in the Brazilian Amazon. Performance reflects preliminary, descriptive point estimates from an initial diagnostic accuracy assessment and should be interpreted with caution; the tool is not intended for standalone diagnosis. Next steps include prospectively powered clinical validation, usability refinements, and regulatory evaluation, including alignment with ANVISA requirements in Brazil. Overall, this work represents an initial step toward closing the gap in clinically supported diagnostic tools for neglected tropical diseases in resource-constrained settings.

## Author summary

Cutaneous leishmaniasis is a major public health challenge in the Brazilian Amazon. Delays in diagnosis increase illness, stigma, and costs, especially in remote communities with limited connectivity. Community health workers and primary care teams are often the first to see suspicious skin lesions, but they lack point-of-care tools to support early triage. We developed and evaluated the feasibility of a mobile application that works entirely offline and uses artificial intelligence to assist with early triage from smartphone photographs of skin lesions. The model was trained on clinical images collected by partner institutions in the Amazon region and integrated into an app optimized for low-resource settings. In a multicenter assessment that included both more standardized (“ideal-world”) images and routine field conditions, the tool identified most cutaneous leishmaniasis cases, returned a result in under a minute, and was well accepted by users. Specificity was moderate, which is appropriate for a triage tool designed to prompt timely referral rather than to replace clinical and laboratory diagnosis. This feasibility study shows that an offline, AI-assisted approach can help accelerate referral and access to care in underserved regions. Next steps include larger, prospective clinical validation, usability refinements based on field feedback, and regulatory evaluation aligned with Brazilian requirements (ANVISA). The same strategy could be adapted to other dermatologic conditions and neglected tropical diseases in similar settings.

## Introduction

Leishmaniasis is a significant neglected tropical disease caused by protozoa of the *Leishmania* genus and transmitted by female phlebotomine sandflies. Cutaneous Leishmaniasis (CL), the most common form, presents as ulcers, papules, or nodules, primarily on exposed skin [1]. It remains a persistent public health challenge, with Brazil being among the 11 countries accounting for 90% of global CL cases [2]. In the Amazon, leishmaniasis parasite transmission persists due to forest exploitation driven by economic interests, with incidence shaped by environmental and economic factors [3].

Delayed diagnosis of CL can lead to secondary infections, disfigurement, self- and social stigma, and impairment of nasal and oropharyngeal function, depending on host-parasite interaction mechanisms [4,5]. It also contributes to the ongoing transmission of the disease and may necessitate prolonged, complex treatments, increasing the risk of medication side effects and escalating healthcare costs, ultimately posing a public health challenge [1,4,6].

In Brazil, community health workers serve as the primary point of access to the healthcare system, facilitating patient referrals to physicians for diagnosis and treatment when medical attention is required, for example, in cases of suspected CL [7]. Integrating new technologies into this setting can enhance the screening and referral process, shorten the time to appropriate treatment, improve healthcare outcomes, and optimize resource utilization [8].

Artificial intelligence (AI) is increasingly valued in healthcare for replicating human cognitive functions. Machine learning (ML), a subfield of AI, utilizes approaches such as deep learning (DL) algorithms to extract patterns from data and perform complex tasks. These techniques are particularly effective in image analysis, as they can identify features such as edges and textures, and intricate patterns with high precision [9,10].

In dermatology, AI has been extensively studied for applications in skin cancer, alopecia, and inflammatory disorders [9–11]. However, its use in neglected tropical diseases remains underexplored, with only seven studies reported using AI to support CL diagnosis. Four studies utilize clinical images [12–14], and three utilize microscopic images [15–17]. Despite its potential, AI model development faces important challenges, including difficulties in real-world validation due to variations in image quality and patient demographics. Generalizability is limited, as models may underperform on new or diverse datasets. Potential biases in training data, such as overrepresentation of certain lesion types or skin tones, can affect diagnostic accuracy for underrepresented groups. Additionally, poor interpretability of deep learning models can limit clinician trust and hinder clinical adoption [11].

To address the gap in early CL diagnosis in remote areas, we conducted a feasibility study to develop and perform an initial diagnostic accuracy assessment of an AI-assisted tool in the Brazilian Amazon. The specific objectives of this study were to: (i) develop an AI model for CL identification using clinical skin lesion images; (ii) integrate the developed AI model into a mobile application, optimized for offline operation in resource-limited settings; (iii) perform initial clinical validation of both the application and the AI model in real-world clinical settings, focusing on assessing their potential performance and acceptability within a triage context.

## Materials and methods

### Ethical statement

This exploratory, applied, retrospective study, focused on technological development, was approved by the Ethics Committee of Albert Einstein Hospital (Approval Number 75460623.5.0000.0071). The study included participants aged 18 years or older, and, when applicable to the study stage, informed consent was obtained through a written Free and Informed Consent Form.

### Sample size

Due to the inherent logistical, ethical, and practical challenges associated with collecting high-quality, labeled clinical data for CL in multicenter, field-based settings within the Brazilian Amazon, a formal a priori power analysis was not conducted. This approach aligns with the current literature, which highlights the empirical nature of sample size determination in AI healthcare studies, often relying on post hoc analyses of learning curves or evaluation of model performance with progressively increasing data [18]. In line with this, prior proof-of-concept work on AI for skin-related NTDs by Yotsu et al. [19] and Barbieri et al. [20] for leprosy also prioritized feasibility, relying on the available image pool rather than targeting a predetermined power, given the acknowledged difficulties in acquiring large, diverse datasets for neglected tropical diseases. Instead, our sample size was determined by available resources and informed by established initial benchmarks from comparable studies in medical imaging. Learning-curve modeling in medical image classification has shown that thousands of images per class may be needed to approach very high accuracy [21]. These benchmarks often suggest around 1,000 images for robust preliminary analyses, acknowledging the significant constraints in acquiring large multimodal datasets typical for NTDs.

### AI model development

The clinical images used for AI model development were obtained from the institutional archives of two partner institutions with extensive expertise in diagnosing skin lesions: the Fundação de Medicina Tropical Doutor Heitor Vieira Dourado (FMT-HVD) and the Fundação Hospitalar Alfredo da Matta (FUHAM), both located in the Amazon region. As referral centers not only for infectious but also for inflammatory, neoplastic, traumatic, and other types of skin lesions, these institutions provided images highly representative of the diverse differential diagnoses encountered in real-life clinical practice.

The data used for model development were derived from previous studies whose ethical approvals explicitly allowed the reuse of these data in subsequent research projects, provided that each new project received approval from the relevant ethics committee and was reviewed by the participating institutions. All data reuse complied with the original scientific objectives and adhered to privacy protection standards. This protocol was submitted to our Ethics Committee and reviewed by the participating institutions.

The dataset comprised smartphone-captured images of leishmaniasis and its differential diagnoses. Leishmaniasis was diagnosed using direct parasitological methods—which may include biopsies, cultures, scrapings—or qPCR when necessary, whereas other dermatologic conditions were diagnosed via histopathology, cultures, or expert clinical evaluation when the presentation was sufficiently characteristic.

**Fig 1:**
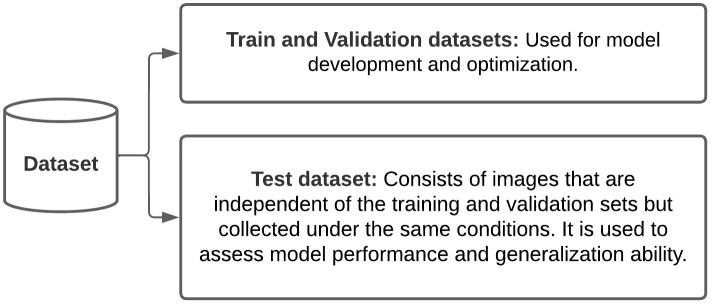
Strategy for dataset division in AI experiments.

Exclusion criteria included mucosal or scalp lesions, those altered by treatments or biopsy, poorly lit images, and duplicates. Mucosal lesions were excluded due to anatomical differences from skin, leading to distinct lesion characteristics that were not the focus of this study. Scalp lesions were excluded because hair shafts interfere with lesion morphology visualization in a single photograph. All images were de-identified and securely transferred per data protection regulations. Additional images from Hospital Israelita Albert Einstein followed the same ethical and inclusion/exclusion criteria. Images were saved in JPEG/PNG and randomly split into training, validation, and test sets (Fig 1). All images were resized to 256×256 pixels and normalized prior to training.

Two deep learning models were implemented for the AI-based CL model: (i) a Segmentation Model to isolate skin lesions and (ii) a Classification Model to identify CL, both developed using Python and TensorFlow.

The Segmentation Model, based on the DeepLab family algorithm [22], isolates lesions to facilitate classification. A dermatologist annotated images using COCO Annotator [23], marking the areas affected by the lesion. This model was trained using a combination of Binary Crossentropy and Dice Loss, optimized with the Adam optimizer (learning rate = 0.001), and early stopping was applied to prevent overfitting.

The Classification Model predicts whether a lesion is CL, processing segmented images from the Segmentation Model. Thirteen deep learning architectures were tested (see S1 Appendix for details). The classification model used Binary Crossentropy loss and was also optimized with Adam.

To further enhance the workflow, a Blur Classification Model was developed to identify blurry images and prompt users to resubmit clearer photographs prior to analysis by the Segmentation and Classification Models (see S2 Appendix for details).

### Mobile application

A mobile application was chosen for the implementation of AI because, in resource-limited settings, mobile phones are often the most accessible and cost-effective technology. The mobile application, developed in React Native, embeds the final AI model in TensorFlow Lite format for offline use. It leverages Java (Android) and Swift (iOS) for cross-platform compatibility and performance optimization (see S3 Appendix for details).

**Fig 2:**
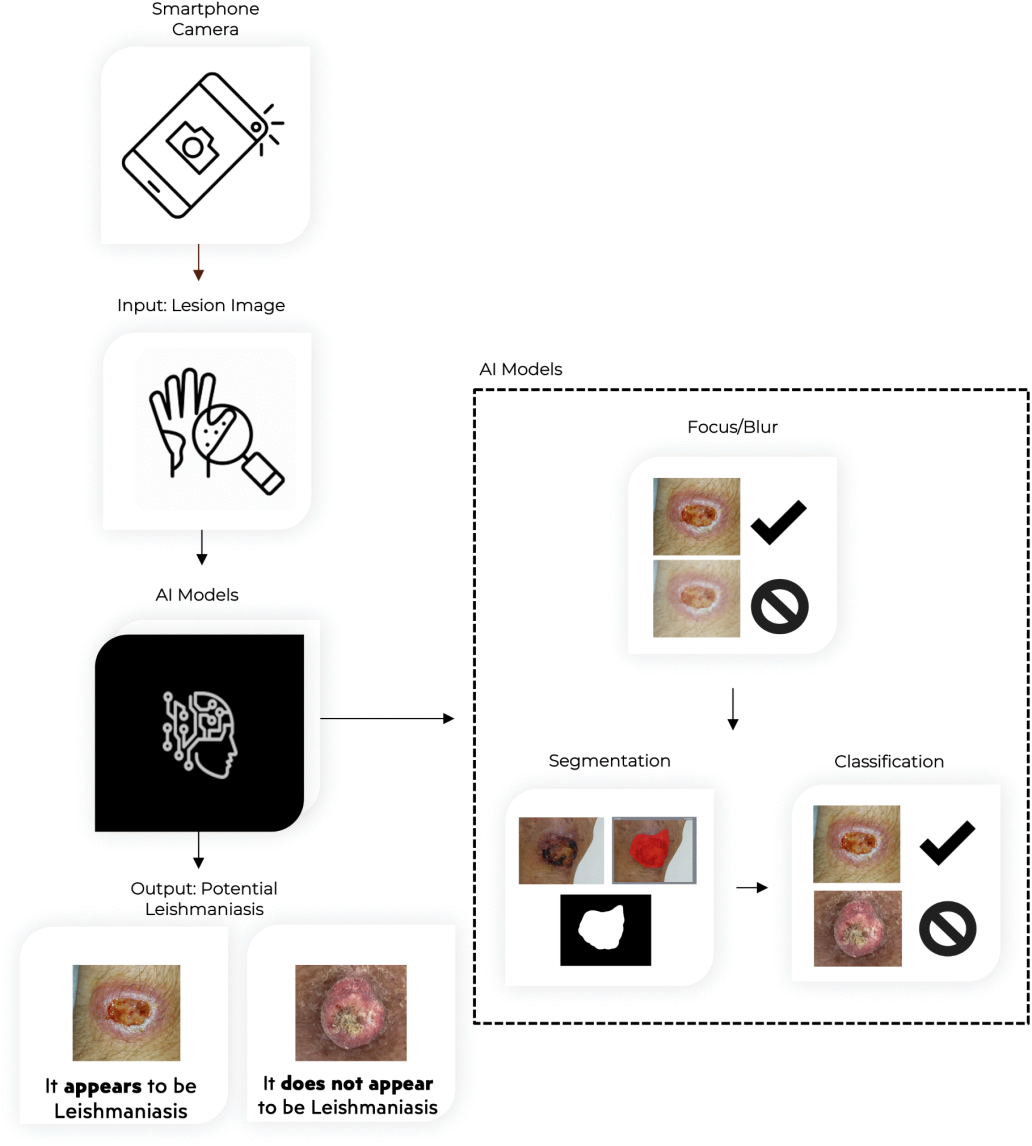
Workflow of the mobile application.

The user interface was designed with simplicity and accessibility in mind, taking into account the needs of healthcare professionals operating in low-resource settings. Users can upload images from the gallery or capture them via the camera. The image is processed by the Blur Classification Model, which prompts re-upload if classified as blurry. Then, the image is processed by the Segmentation and Classification Models, generating one of two results: “Appears to be leishmaniasis” or “Does not appear to be leishmaniasis” (Fig 2).

Additionally, users enter patient information and select the lesion’s location on an avatar. This data is recorded for the final report but is not included in AI analysis. Privacy policies and terms of use are accessible in the app, and all patient data is securely handled in compliance with data protection regulations. The application includes features such as consultation history for tracking previous analyses. The app was developed in Brazilian Portuguese.

### Multicenter validation

Between November 2024 and January 2025, the mobile application was tested in clinical settings. Seven smartphones (Moto G34 for Android and iPhone 11 for iOS) were provided to two reference centers in the Amazon (FMT-HVD and FUHAM) for use by healthcare professionals and community health workers. Adults (≥18 years) with suspected CL or differential diagnoses who provided informed consent were included. Exclusion criteria comprised mucosal or scalp lesions, those altered by treatment or biopsy, poorly lit images, and duplicates.

For each analyzed lesion, the smartphone used, the AI prediction, and the final diagnosis established by the specialist were recorded. The final diagnosis was based on anamnesis and laboratory tests (histopathology, direct examination of lesion scrapings, culture, and qPCR), as needed. The application’s performance was assessed under two conditions: (i) Real-world scenario—all skin lesion images meeting inclusion criteria were considered, and (ii) Ideal-world scenario—the analysis was restricted to cases involving diagnoses the AI model had been exposed to during its development.

To statistically assess whether the AI model’s predictions were significantly associated with the reference diagnoses, we performed a Chi-square test [24] of independence for each scenario. This test evaluated whether the distribution of AI predictions differed significantly between true diagnostic categories (Leishmaniasis vs. Other diagnoses), using the resulting confusion matrices as input.

Additionally, user feedback was collected through standardized REDCap questionnaires, allowing for anonymous responses. The questions addressed navigation ease, challenges encountered, response time, usage frequency, triage and referral assistance, and suggestions for improvement.

### Statistical analysis

The AI models were comprehensively evaluated using a set of standard metrics. For the Classification Model and the full AI pipeline, these included sensitivity, specificity, positive predictive value, accuracy, F1-score, Area Under the Receiver Operating Characteristic Curve (AUC-ROC), and odds ratio. The Segmentation Model’s performance was specifically assessed using the Dice coefficient, a widely recognized agreement metric for evaluating the spatial overlap between predicted and expert reference segmentation masks, and mean Average Precision (mAP) [25–27]. (see S4 Appendix for details).

This work was designed as an early-stage feasibility study. The following hypotheses were formulated: (i) H1a (Classification Model accuracy): The accuracy of the Classification Model for identifying CL is significantly greater than 0.50 (chance level); H0a: The accuracy of the Classification Model for identifying CL is less than or equal to 0.50; (ii) H1b (Full AI Pipeline AUC-ROC): The AUC-ROC of the full AI pipeline (Segmentation + Classification) for identifying CL is greater than 0.70; H0b: The AUC-ROC of the full AI pipeline for identifying CL is less than or equal to 0.70; (iii) H1c (Multicenter Validation - Ideal-world Scenario Sensitivity): The sensitivity of the full AI pipeline in the multicenter validation (ideal-world scenario) is greater than 0.75; H0c: The sensitivity of the full AI pipeline in the multicenter validation (ideal-world scenario) is less than or equal to 0.75.

## Results

### AI models

For AI model development, a total of 1,290 images were provided by partner institutions. After applying eligibility criteria, 1,224 images were included in the final dataset, evenly split between 612 CL cases and 612 other diagnoses, including sporotrichosis, skin cancer, actinic keratosis, venous ulcer, neuropathic ulcer, pressure ulcer, arterial ulcer, diabetic foot ulcer, and lymphoma.

**Fig 3:**
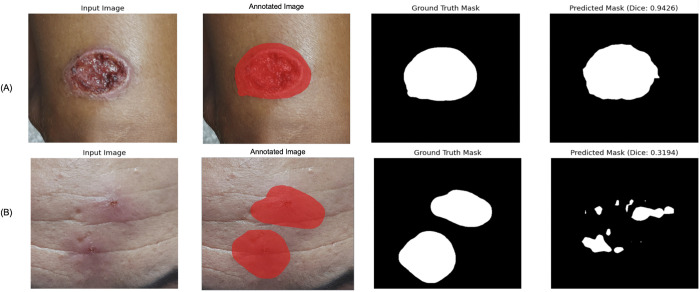
Examples of input images, manually annotated images, ground truth masks, and predicted masks. A: High-performing case (Dice = 0.94). B: Low-performing case (Dice = 0.32).

For the Segmentation Model, of the 1,224 images, 200 were randomly assigned to the test set, while 1,024 were used for training and validation (80:20 stratified split) [28]. The model was implemented using DeepLabV3 [22] with a MobileNetV2 [29] backbone. It achieved a mean Average Precision (mAP) of 0.83 and a Dice coefficient of 0.80. Fig 3 presents examples of segmentation performance, showcasing both high-performing and low-performing cases.

For the Classification Model to identify CL, of the 1,224 images, 64 randomly assigned to the test set, while 1,160 (evenly split between ‘leish’ and ‘not-leish’) were used for training and validation (80:20 stratified split) [28]. It achieved the best performance with DenseNet121 architecture [30], with an accuracy of 0.88.

### Mobile application

**Fig 4:**
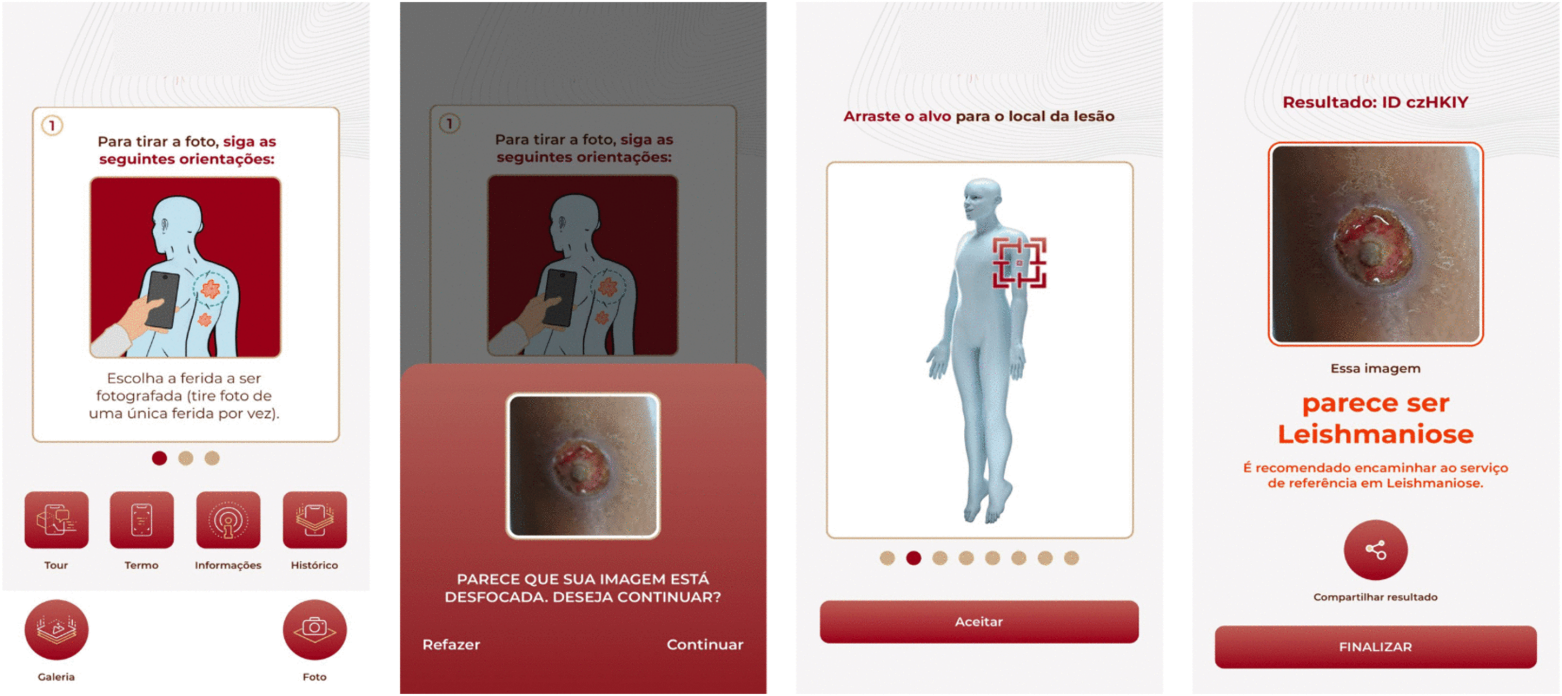
Interfaces of the mobile application.

**Fig 5:**
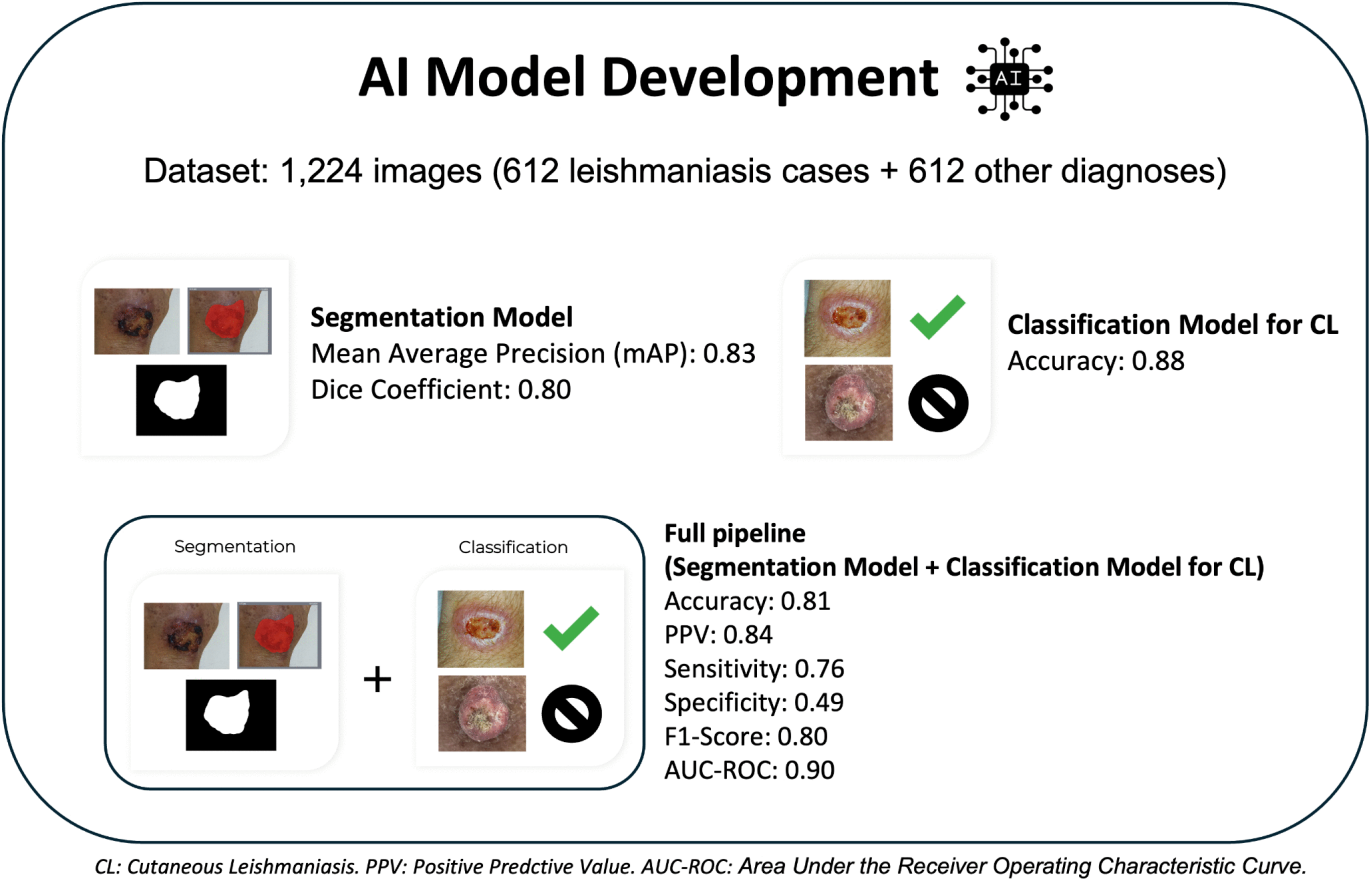
Summary of the performance achieved by the individual models and the entire pipeline.

The application presents an intuitive, user-friendly interface (Fig 4). The combined performance of the Segmentation and Classification Models achieved an accuracy of 0.81, a positive predictive value of 0.84, sensitivity of 0.76, specificity of 0.49, F1-score of 0.80, and AUC-ROC of 0.90. Fig 5 summarizes the performance of individual models and the full AI pipeline.

### Multicenter validation

The multicenter validation included 217 participants (≥ 18years) who met eligibility criteria and provided informed consent. More than one lesion could be assessed per participant, totaling 386 analyzed lesions. Thirty-two duplicate images, three low-quality images, and 145 images without a final diagnosis were excluded, leaving 206 lesions for real-world analysis. Among these, 92 were leishmaniasis cases, while 114 had other diagnoses.

**Fig 6:**
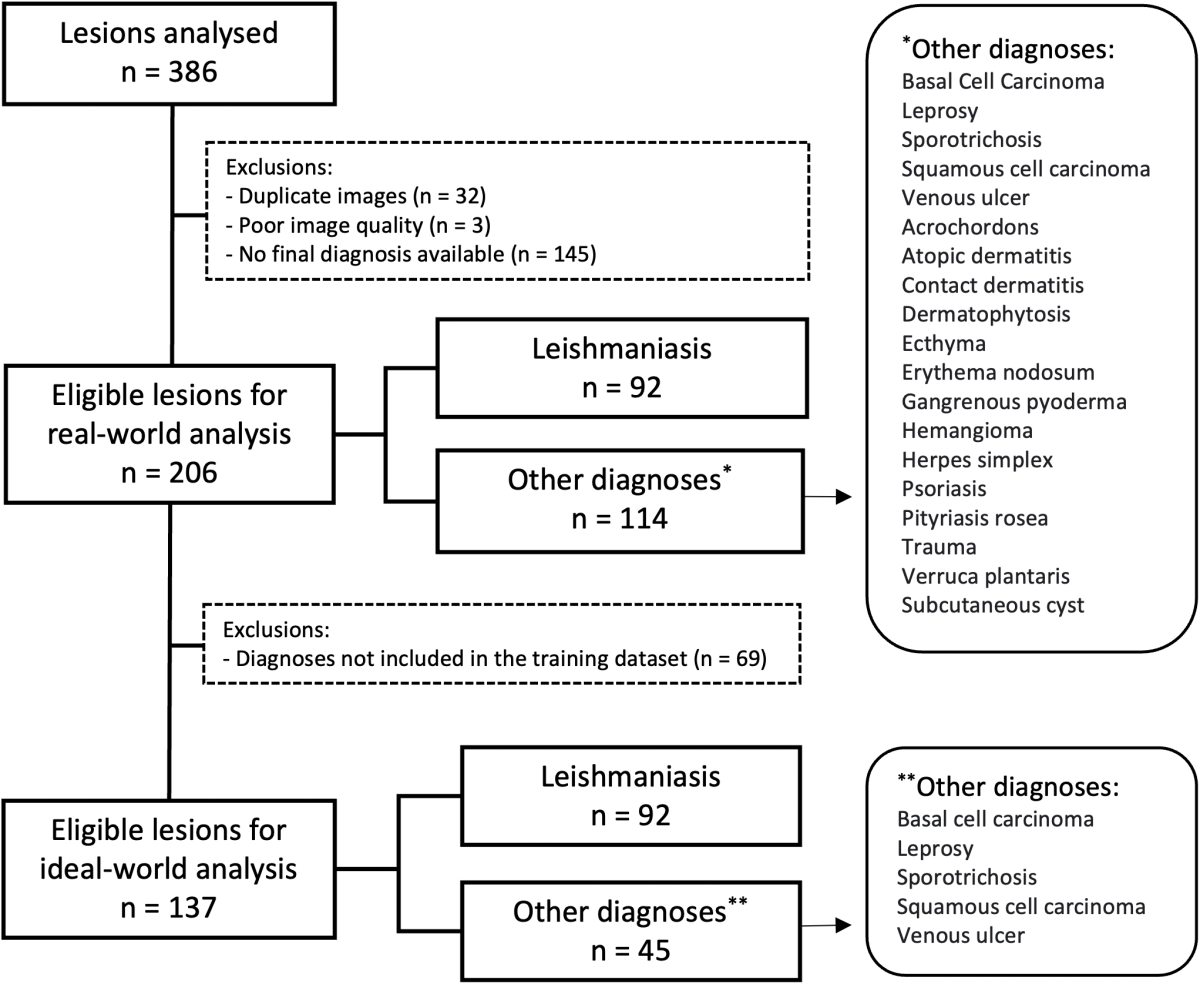
Flowchart of the data analysis process, showing lesion selection for the real-world and ideal-world analyses, along with the included diagnoses.

For the ideal-world analysis, 69 cases with diagnoses not included in the training phase were excluded, leaving 137 lesions, of which 92 were leishmaniasis cases and 45 had other diagnoses. Fig 6 illustrates the flowchart of the multicenter validation data analysis.

In the real-world analysis, the AI correctly identified 85 out of 92 leishmaniasis cases, while 7 were misclassified as negative. Among 114 other diagnoses, 74 were incorrectly classified as leishmaniasis, and 40 were correctly identified as negative. The model achieved a sensitivity of 0.92, specificity of 0.35, accuracy of 0.61, an F1-score of 0.68, and an odds ratio of 6.56.

In the ideal-world analysis, the AI correctly identified 85 out of 92 leishmaniasis cases, with 7 misclassified as negative. Among 45 other diagnoses, 26 were misclassified as leishmaniasis, while 19 were correctly classified as negative. The model achieved a sensitivity of 0.92, specificity of 0.42, accuracy of 0.76, an F1-score of 0.84, and an odds ratio of 8.87.

Table 1 presents the confusion matrices, while Table 2 summarizes both analyses.

**Table 1:** Confusion matrices for real-world and ideal-world analyses.

Analysis type	Predicted Diagnosis	AI suggests Leishmaniasis	AI does not suggest Leishmaniasis	Total
**Real-world**	Leishmaniasis	85	7	92
	Other diagnoses	74	40	114
	Total	159	47	**206**
**Ideal-world**	Leishmaniasis	85	7	92
	Other diagnoses	26	19	45
	Total	111	26	**137**

**Table 2:** Multicenter validation of the AI model: Real-world and Ideal-world analyses.

	Real-world analysis	Ideal-world analysis
**Images**	206 (92 leishmaniasis + 114 other diagnoses*)	137 (92 leishmaniasis + 45 other diagnoses**)
**Sensitivity**	0.92	0.92
**Specificity**	0.35	0.42
**Accuracy**	0.61	0.76
**F1-score**	0.68	0.84
**Odds ratio**	6.56	8.87

*Some diagnoses not included in the AI model development phase were also present, requiring the model to analyze cases it had never encountered before.

**Only diagnoses included in the AI model development phase were considered.

In both scenarios, the association between the AI predictions and the reference diagnoses was statistically significant, indicating that the model’s performance was not due to chance. In the real-world analysis, the chi-square test yielded a value of $\chi^{2}$ = 21.83 with 1 degree of freedom and a p-value of 3.0 × 10^−6^. Similarly, in the ideal-world analysis, the result was $\chi^{2}$ = 23.55 with a p-value of 1.0 × 10^−6^. These results confirm a significant relationship between the model’s output and the ground-truth diagnoses in both evaluation contexts.

Feedback was collected from seven community health workers and healthcare professionals, all of whom reported daily use of the application. Regarding usability, four participants rated the application as ‘very easy’ to use, two as ‘easy’, and one as ‘average’. Six participants reported no difficulties in application use, while one reported difficulty reading text against a dark background. Processing time was reported as ‘less than one minute’ by six participants and as ‘one to two minutes’ by one participant. In evaluating the application’s impact on triage and referral processes, three participants noted it ‘slightly facilitated’ these activities, three reported it ‘moderately facilitated’, and one stated it ‘did not facilitate’ the process. Suggestions for future improvements included integrating epidemiological data and providing guidance on photo standardization.

## Discussion

### Overview of existing research

Among the seven AI-based studies for CL diagnosis described in the literature, three utilize microscopic images [15,16,17], supporting diagnosis after laboratory testing. While valuable, their applicability in resource-limited endemic areas is constrained by the availability of laboratory infrastructure.

One study utilizes skin lesion images, applying machine learning to differentiate CL, Buruli ulcer, and leprosy. While useful in specific settings, its scope is limited to preselected diseases, whereas real-world differential diagnosis involves a broader range of conditions.

The remaining three studies, like ours, use deep learning models for CL diagnosis based on clinical skin lesion images. While they provide valuable insights, their findings should be interpreted with caution due to methodological differences, dataset variability, and contextual challenges. Table 3 presents an overview of these studies, highlighting their key limitations.

**Table 3:** Summary of studies applying deep learning to identify CL in clinical images and their key limitations.

Article (Author, title, journal, year)	Objective	Model Development	Key Limitations
- Leal et al. [12] - Automated identification of Cutaneous Leishmaniasis lesions using deep-learning-based artificial intelligence - Biomedicines - 2024	To evaluate the performance of the AlexNet algorithm in identifying CL lesion images.	- Total images: 2458 - CL cases: 1787 (73% of total) - Other dermatoses: 671 (27% of total) - Camera type: Professional camera - Accuracy: 95%	The reported accuracy is likely overestimated due to an imbalanced dataset.
- Noureldeen et al. [13] - Deep learning model for Cutaneous Leishmaniasis detection and classification using Yolov5 - African Journal of Advanced Pure and Applied Sciences - 2023	To develop a deep learning model based on the YOLO network for detecting and classifying CL ulcers in photos of suspected CL cases.	- Total images: not specified - CL cases: 160 - Other dermatoses: not specified - Camera type: Mobile phone camera - Accuracy (average): 70%	The reported accuracies may not reflect the model’s actual performance due to the lack of details regarding dataset composition.
- Arce-Lopera et al. [14] - Presumptive diagnosis of Cutaneous Leishmaniasis - Frontiers in Health Informatics - 2021	To develop a mobile application to aid pre-diagnosis of CL using an automatic image recognition software based on a convolutional neural network model.	- Total images: 2022 - CL cases: not specified - Other diagnoses: not specified - Camera type: not specified - Accuracy: 93%	The reported accuracy may not represent the model’s performance due to unclear dataset composition. Additionally, inconsistencies exist between the reported number of cases and accuracy values in the text and table.

CL: Cutaneous leishmaniasis. YOLOv5 and AlexNet are deep learning architectures.

The study by Leal et al. [12] used an imbalanced dataset, which can bias models toward the majority class. For instance, if 90% of images are CL cases, a model always predicting ‘leishmaniasis’ would achieve 90% accuracy without effectively distinguishing other conditions. This limitation suggests that the reported 95% accuracy may not reflect actual performance. Additionally, their model was trained on high-resolution images from a professional camera, requiring specialized equipment not readily available in low-resource environments, whereas our approach uses smartphone images to improve accessibility for community health workers in remote areas.

The studies by Noureldeen et al. [13] and Arce-Lopera et al. [14] lack crucial dataset details, such as the number of images per class and the specific differential diagnoses included. This information is critical, as distinguishing CL from diseases with distinct lesions (e.g., blisters) is simpler than differentiating from conditions with similar clinical presentations (e.g., skin cancer and sporotrichosis). Our study incorporates these challenging differential diagnoses, making our model clinically relevant. Furthermore, inconsistencies in dataset composition and reported accuracy raise concerns about the validity of results in Arce-Lopera et al. [14].

Overall, the literature review underscores a lack of comprehensive studies on deep learning to identify CL, limiting direct comparisons. Our study addresses these gaps by using a balanced dataset, evenly distributed between CL and differential diagnoses, ensuring a more reliable and generalizable model.

### AI model performance

The Segmentation Model achieved a mAP of 0.83 and a Dice coefficient of 0.80, indicating strong overlap between predicted masks and ground truth, highlighting its reliability in lesion delineation. However, some Dice scores were lower due to poorly defined boundaries or image quality variations. Specifically, mis-segmentations—such as incomplete lesion contours or inclusion of background regions—may introduce noise or irrelevant features into the classification step, potentially lowering diagnostic accuracy. Thus, optimizing the segmentation model would likely reduce such errors and help improve the reliability and specificity of the entire diagnostic process.

The Classification Model to identify CL achieved an accuracy of 0.88, demonstrating strong potential for aiding in CL case identification. Direct comparisons with previous studies are not feasible due to limited details on dataset composition and methodological differences, as discussed earlier.

The full AI pipeline (Segmentation + Classification Models) demonstrated strong performance, achieving moderate-to-high sensitivity (0.76), high accuracy (0.81), an F1-score of 0.80, and an AUC-ROC of 0.90, reflecting robust performance across key metrics. Specificity was low (0.49), which aligns with the model’s intended application as a triage tool. Given its design, the model prioritizes sensitivity, in order to minimize false negatives, which results in some false positives and, consequently, lower specificity. Notably, false-positive cases would still be reviewed by a physician, who can establish the final diagnosis with complementary tests. This approach ensures patients are not underdiagnosed and supports timely medical assessment in clinical practice.

Compared to the gold standard, Direct Parasitological Diagnosis—which can be performed through biopsies, cultures, scrapings, or impression smears—our AI-based tool serves as a complementary approach. While the gold standard offers high specificity, its sensitivity remains a concern [31], whereas our model prioritizes sensitivity over specificity. Rather than replacing the gold standard, the AI model functions as an initial screening tool at the first point of contact in the healthcare system, requiring neither specialized personnel nor sophisticated tests, facilitating early detection in resource-limited settings.

### Multicenter validation

In the ideal-world analysis, where only diagnoses included in the training phase were considered, the metrics seem aligned with the development phase, demonstrating its consistency when applied to data similar to the training set (sensitivity of 0.92, an F1 score of 0.84, an accuracy of 0.76, and a specificity of 0.42).

Notably, the ideal-world analysis exhibited data imbalance due to the use of a non-probabilistic, convenience-based sampling method. However, the odds ratio (OR), which measures the likelihood of correct predictions within each group, is less affected by class proportions. With 85 true positives, 19 true negatives, 26 false positives, and 7 false negatives, the OR was 8.87, indicating that the odds of a correct prediction were 8.87 times higher than those of a misclassification. Since the OR is based on the ratio of correct to incorrect predictions rather than the overall class distribution, it remains robust to class imbalance, thereby supporting the application’s predictive capability for leishmaniasis detection.

In this analysis, most misclassifications were false positives. The highest false positive rates were observed for leprosy and sporotrichosis, whereas venous ulcer and skin cancer showed the lowest rates. Specifically, when normalized by class size, false-positive rates exceeded 50% for leprosy and sporotrichosis. Skin cancer exhibited a false-positive rate of approximately one third of cases, whereas no false positives were observed for venous ulcers. Interpretation is limited by the small sample size per diagnosis. This pattern may be explained by the marked clinical similarity between sporotrichosis and leishmaniasis, as well as the broad clinical spectrum exhibited by leprosy. The model tends to classify lesions with previously unseen patterns as positive, which may account for the high false positive rate observed for leprosy, given its diverse presentations of primary lesions. An area for improvement involves expanding the dataset with additional images of sporotrichosis and leprosy, especially across the diverse clinical presentations of leprosy, as these diagnoses are most frequently confused by the model.

In real-world analysis, sensitivity remained high at 0.92, while specificity, F1 score, and accuracy were lower at 0.35, 0.68, and 0.61, respectively. This decline occurred because the model, which was developed to detect potential leishmaniasis cases, had been trained primarily on common differential diagnoses of leishmaniasis. However, real-world validation included lesion types not previously encountered by the model during the training phase. As mentioned before, when faced with unseen images, the model tends to classify them as positive due to its triage design, affecting performance.

In real-world analyses, non-papulo-ulcerative lesions—such as bullae, cysts, and erythematous-desquamative plaques—demonstrated a higher false positive rate compared to papulo-ulcerative lesions (68.8% versus 21.3%). This finding may reflect the composition of the training dataset, which predominantly included papulo-ulcerative lesions representative of leishmaniasis and its differential diagnoses. Although false positives increase the workload for confirmatory testing, this is aligned with the model’s objective of minimizing false negatives, thereby prioritizing patient safety. To enhance the model’s performance, two approaches can be tested: expanding the training dataset to include a broader variety of lesion types, and providing clearer guidelines to users about which cases are suspected of leishmaniasis and therefore should be analyzed by the model. The feedback from community health workers and healthcare professionals underscores the application’s relevance in clinical workflows, demonstrating its usability, efficiency, and practicality in demanding settings. While the tool was well-received, suggestions for improvement—such as integrating epidemiological data and providing guidance on photo standardization—could further strengthen its acceptance and facilitate its incorporation into clinical practice.

Our findings met the predefined performance benchmarks established for this feasibility study. H1a, positing a Classification Model accuracy significantly greater than chance level (0.50), was affirmed by the observed accuracy of 0.88. Given the balanced classes, these results are consistent with the model capturing discriminative signals to distinguish CL from other conditions in this dataset. Furthermore, the full AI pipeline’s discriminative power, as assessed by H1b (AUC-ROC > 0.70), was supported, achieving an AUC-ROC of 0.90. This suggests robust performance when both segmentation and classification components are integrated. Finally, H1c, which targeted a sensitivity greater than 0.75 in the ideal-world multicenter validation, was also met, with the model demonstrating a sensitivity of 0.92. This high sensitivity confirms the AI’s potential to correctly identify true CL cases, aligning with its design as a triage tool prioritizing the minimization of false negatives. Collectively, the confirmation of these hypotheses indicates the AI-powered mobile app’s technical viability and initial clinical utility for CL diagnosis, supporting its potential as a valuable aid for healthcare providers in resource-limited settings.

The use of smartphone-acquired images and offline functionality enhances the application’s applicability in remote areas, such as communities in the Amazon region. Furthermore, it enables scalability to other leishmaniasis-endemic regions in Brazil and globally, where healthcare access is limited.

Studies have shown that patients perceive medical photography as enhancing their healthcare experience and often recommend its use to others [32,33]. These findings highlight the impact of medical photography—independent of AI—on patients’ perceptions of care. We propose that capturing an image of a skin lesion, combined with AI analysis, could further strengthen this perception. A major challenge in remote areas with limited healthcare access is the tendency of patients to underestimate the severity of their condition or fail to seek medical attention in a timely manner. Such delays often result in advanced-stage diagnoses, whether for infectious diseases like leishmaniasis, neoplastic conditions such as skin cancer, or other serious illnesses. In this context, an AI-powered application analyzing a patient’s skin lesion photograph could encourage patient engagement in the diagnostic and treatment process. In this scenario, lower specificity is less concerning, as the application remains relevant regardless of the diagnosis. While the goal is to develop a highly accurate model for CL identification, the current model still holds value for this purpose.

Future efforts should focus on increasing the number of images and diversifying the training dataset to include a broader range of diagnoses, ensuring greater lesion diversity and improving generalizability. Incorporating metadata—such as endemic region, lesion location, duration, and symptoms (e.g., pain, pruritus, or asymptomatic presentation)—could refine diagnostic accuracy. Additionally, developing a structured scoring system that integrates metadata, as proposed by Rubiano et al. [34], may further enhance AI-powered CL diagnosis, integration of such variables may enhance performance and robustness by reducing reliance on image data alone. Systematic collection of user feedback through in-app surveys and feedback forms could also be implemented to guide iterative improvements to both the app’s interface and the AI model’s performance.

## Conclusion

To our knowledge, this is the first AI-based model for CL identification that operates entirely offline, trained on a balanced dataset of CL and differential diagnosis images from the Brazilian Amazon. By functioning on-device, the tool preserves patient privacy, avoids dependence on internet connectivity, and is suitable for remote, resource-limited settings. In clinical practice, this tool has the potential to support frontline healthcare providers in the early identification and referral of suspected CL cases, streamlining triage workflows, accelerating diagnostic pathways, and optimizing the allocation of scarce healthcare resources.

Limitations should be acknowledged. First, the model demonstrates relatively low specificity, reflecting a deliberate design choice to prioritize sensitivity in a triage-oriented context rather than definitive diagnosis. While this approach may increase the number of false-positive cases, such cases are expected to be resolved during subsequent specialist evaluation, thereby reducing the risk of missed diagnoses and supporting timely referral and care.

Additional challenges give space for further investigation. The generalizability of model performance may vary across geographical regions, healthcare settings, imaging devices, and patient populations, underscoring the importance of external validation in independent cohorts. Performance may also be enhanced by expanding the training dataset to include a broader spectrum of dermatological conditions and by providing clearer guidance to users regarding lesion types commonly associated with suspected leishmaniasis. Prospective data collection across diverse clinical and epidemiological contexts will be essential to strengthen robustness and clinical applicability.

We reaffirm that this is a feasibility study focused on preliminary performance in this kind of tool. Accordingly, we report descriptive point estimates, with precision to be formally quantified in future, prospectively powered analyses. Future phases of this work will include clinical validation, regulatory evaluation, and alignment with ANVISA requirements in Brazil. Overall, this study represents an initial step toward addressing the critical gap in clinically supported diagnostic tools for neglected tropical diseases, particularly in resource-constrained settings.

## Supporting information

S1 Appendix Classification Model for Cutaneous Leishmaniasis. (PDF)

S2 Appendix Classification Model for Blur in Skin Lesion Image. (PDF)

S3 Appendix Mobile application. (PDF)

S4 Appendix Performance metrics. (PDF)
