# Supplementary material for: Feasibility study of an AI-powered mobile app to support cutaneous leishmaniasis diagnosis in the Brazilian Amazon
Source: PLoS Negl Trop Dis. 2026 May 27;20(5):e0014313. doi: 10.1371/journal.pntd.0014313 (PMC13215602; doi:10.1371/journal.pntd.0014313)
Supplement: S1 Appendix — (PDF) [file pntd.0014313.s001.pdf]

S1 Appendix - Classification Model for Cutaneous Leishmaniasis

Title: AI-Powered Mobile App to Support Cutaneous Leishmaniasis Diagnosis in the Brazilian Amazon

Classification Model for Cutaneous Leishmaniasis

Thirteen state-of-the-art deep learning architectures were implemented, including MobileNet, MobileNetV2, MobileNetV3Small, MobileNetV3Large, ResNet50, ResNet50V2, EfficientNetV2M, EfficientNetV2S, InceptionV3, VGG16, DenseNet121, DenseNet169, and MobileViT[1]. Among these, DenseNet121 achieved the best performance, with 0.88 validation and 0.96 test accuracy, outperforming all other models, which remained below 85% accuracy. Its compact size (7.15 MB) also contributed to its selection, offering an optimal trade-off between accuracy and efficiency.

References

1. Team K. Keras documentation: Keras Applications [Internet]. [cited 2025 Feb 1]. Available from: https://keras.io/api/applications/
