# Supplementary material for: Feasibility study of an AI-powered mobile app to support cutaneous leishmaniasis diagnosis in the Brazilian Amazon
Source: PLoS Negl Trop Dis. 2026 May 27;20(5):e0014313. doi: 10.1371/journal.pntd.0014313 (PMC13215602; doi:10.1371/journal.pntd.0014313)
Supplement: S2 Appendix — (PDF) [file pntd.0014313.s002.pdf]

S2 Appendix - Classification Model for Blur in Skin Lesion Image

Title: AI-Powered Mobile App to Support Cutaneous Leishmaniasis Diagnosis in the Brazilian Amazon

## Classification Model for Blur in Skin Lesion Image

The model was developed using Python and the TensorFlow framework, to identify blurred images, notify users about their low quality, and allow them to upload another image for analysis.

The dataset was composed of 600 skin lesion images from the same dataset for the Segmentation and Classification for CL Models described in the main text. To generate the unfocused class, we simulated the process of deblurring images by applying a Gaussian blur transformation. This method introduced varying levels of blur by averaging pixel values within a defined neighborhood[1]. The transformation was consistently applied across the dataset to simulate defocusing effects. After applying this perturbation, images with a blurriness score ≤ 0 were selected to group unfocused. Of the 600 images, balanced between focused and unfocused classes, 200 images were allocated for testing, while the remaining 400 were split into 80% for training and 20% for validation.

Thirteen state-of-the-art deep learning architectures were implemented, including MobileNet, MobileNetV2, MobileNetV3Small, MobileNetV3Large, ResNet50, ResNet50V2, EfficientNetV2M, EfficientNetV2S, InceptionV3, VGG16, DenseNet121, DenseNet169, and MobileViT[2,3]. The best performance was achieved using the MobileNet architecture[4], which attained an accuracy of 0.96. MobileNet was selected not only for its accuracy but also for its efficiency. Designed for inference on resource-constrained devices, it uses depthwise separable convolutions to significantly reduce the number of parameters and computational cost. After quantization, the model size is only 3.31 MB, making it highly suitable for offline deployment in mobile applications—particularly in low-resource settings such as remote

areas of the Brazilian Amazon. This balance between accuracy and efficiency was a key factor in its selection in this context.

References


1. Hummel RA. Deblurring Gaussian Blur.

2. Team K. Keras documentation: Keras Applications [Internet]. [cited 2025 Feb 1]. Available from: https://keras.io/api/applications/

3. Mehta S, Rastegari M. MobileViT: Light-weight, General-purpose, and Mobile-friendly Vision Transformer [Internet]. arXiv; 2022 [cited 2025 Feb 1]. Available from: http://arxiv.org/abs/2110.02178

4. Howard AG, Zhu M, Chen B, Kalenichenko D, Wang W, Weyand T, et al. MobileNets: Efficient Convolutional Neural Networks for Mobile Vision Applications [Internet]. arXiv; 2017 [cited 2025 Feb 1]. Available from: http://arxiv.org/abs/1704.04861
