# Supplementary material for: Feasibility study of an AI-powered mobile app to support cutaneous leishmaniasis diagnosis in the Brazilian Amazon
Source: PLoS Negl Trop Dis. 2026 May 27;20(5):e0014313. doi: 10.1371/journal.pntd.0014313 (PMC13215602; doi:10.1371/journal.pntd.0014313)
Supplement: S3 Appendix — (PDF) [file pntd.0014313.s003.pdf]

S3 Appendix - Mobile application

Title: AI-Powered Mobile App to Support Cutaneous Leishmaniasis Diagnosis in the Brazilian Amazon

Mobile application

The application was developed using React Native, based on the Expo Bare Workflow.

The AI models were converted into TensorFlow Lite (.tflite) format using the TensorFlow Lite Converter, enabling efficient on-device inference. During conversion, quantization techniques were applied to reduce model size and latency without significantly compromising accuracy. Metadata was incorporated to optimize pre- and post-processing on the device, enhancing both usability and system integration.

The application is compatible with Android devices running version 6.0 (API 23) or later, targeting SDK 14 (API 34), and requiring ARMv7 architecture, 2 GB of RAM, and 100 MB of storage. For iOS, the application supports version 13.4 or higher on devices with ARM64 architecture (iPhone 6S or later), requiring 2 GB of RAM and 150 MB of storage. The application is compatible with Android devices running version 6.0 (API 23) or later, targeting SDK 14 (API 34), and requiring ARMv7 architecture, 2 GB of RAM, and 100 MB of storage. For iOS, the application supports version 13.4 or higher on devices with ARM64 architecture (iPhone 6S or later), requiring 2 GB of RAM and 150 MB of storage.
