# Supplementary material for: Feasibility study of an AI-powered mobile app to support cutaneous leishmaniasis diagnosis in the Brazilian Amazon
Source: PLoS Negl Trop Dis. 2026 May 27;20(5):e0014313. doi: 10.1371/journal.pntd.0014313 (PMC13215602; doi:10.1371/journal.pntd.0014313)
Supplement: S4 Appendix — (PDF) [file pntd.0014313.s004.pdf]

S4 Appendix - Performance metrics

Title: AI-Powered Mobile App to Support Cutaneous Leishmaniasis Diagnosis in the Brazilian Amazon

Performance metrics

Sensitivity, specificity, positive predictive value, accuracy, F1-score, AUC-ROC, mean Average Precision, Dice coefficient, and odds ratio were chosen because they capture different aspects of classification performance that are crucial for assessing the effectiveness and reliability of our AI-based model for CL identification. These metrics collectively allow us to evaluate the model's capability to accurately detect CL cases, distinguish them from non-cases, and generalize to real-world clinical scenarios, which aligns directly with the primary aims of developing, integrating, and validating our AI system in clinical practice. The formulas and definitions for all metrics used in this study are provided below[1,2,3].

For the computation of these metrics, the confusion matrix was utilized:

	Predicted Positive	Predicted Negative
Actual Positive	True Positive (TP)	False Negative (FN)
Actual Negative	False Positive (FP)	True Negative (TN)

1. **Sensitivity**

Sensitivity, also referred to as recall, measures the model's ability to correctly identify individuals who truly have Cutaneous Leishmaniasis. Sensitivity is calculated as follows:

$$\text{Sensitivity} = \frac{TP}{TP + FN}$$

2. Specificity

Specificity, also known as the true negative rate, measures the ability to correctly identify individuals who do not have Cutaneous Leishmaniasis. Specificity is calculated as follows:

$$\text{Specificity} = \frac{TN}{TN + FP}$$

3. Positive Predictive Value (PPV)

PPV quantifies the likelihood that individuals identified as positive by the model truly have Cutaneous Leishmaniasis. PPV is calculated as follows:

$$\text{PPV} = \frac{TP}{TP + FP}$$

4. Accuracy

Accuracy is defined as the proportion of correct predictions—including both true positive and true negative cases—relative to the total number of predictions. Accuracy is calculated as follows:

$$\text{Accuracy} = \frac{TP + TN}{TP + TN + FP + FN}$$

5. F1-score

The F1-score is the harmonic mean of Precision (PPV) and Recall (Sensitivity), balancing the trade-off between identifying all true cases and avoiding false positives. This is especially valuable when class distributions are imbalanced, as is common in clinical datasets, ensuring robust validation of the model's performance. F1-score is calculated as follows:

$$\text{F1-score} = \frac{2 \times \text{Precision} \times \text{Recall}}{\text{Precision} + \text{Recall}}$$

In which Precision is the same as PPV, and is calculated as follows:

$$\text{Precision} = \frac{TP}{TP + FP}$$

And Recall, which is equivalent to Sensitivity, is calculated as follows:

$$\text{Recall} = \frac{TP}{TP + FN}$$

6. AUC-ROC

The ROC curve (Receiver Operating Characteristic) is a plot that represents a classifier's diagnostic capability by displaying its performance across a range of decision thresholds. The area under the ROC curve (AUC) serves as a widely used single-value metric that quantifies this performance. Notably, an AUC score of 0.5 corresponds to the result expected from random guessing. The AUC can be calculated as the area of trapezoids formed between consecutive points on the ROC plot.

$$AUC = 1 - \frac{1}{2}\left(\frac{FP}{FP + TN} + \frac{FN}{FN + TP}\right)$$

7. Mean Average Precision (mAP)

Mean Average Precision (mAP) is a widely adopted metric for evaluating the performance of segmentation and object detection models. It quantifies how accurately a model predicts the spatial extent of a target by assessing the agreement between the predicted and ground truth masks across varying levels of overlap.

In this study, we computed mAP following the COCO evaluation protocol, which averages the model's precision across multiple Intersection over Union (IoU) thresholds, specifically from 0.50 to 0.95 in increments of 0.05. The Intersection over Union (IoU) is defined as:

$$\mathrm{IoU} = \frac{TP}{TP + FP + FN}$$

* TP is the number of true positive pixels (correctly predicted lesion areas),

* FP is the number of false positive pixels (non-lesion areas incorrectly predicted as lesion),

* FN is the number of false negative pixels (lesion areas missed by the model).

For each IoU threshold, predictions are considered correct if the computed IoU between the predicted and ground truth masks meets or exceeds the threshold. Precision and recall are then calculated at different confidence thresholds.

A precision-recall curve is generated by varying the confidence score thresholds, and the Average Precision (AP) is computed as the area under this curve (AUC-PR) for each IoU

threshold. The final mAP score is obtained by averaging the AP values across all IoU thresholds.

$$\text{mAP} = \frac{1}{n}\sum_{i=1}^{n} AP_i$$

8. Dice Coefficient (DC)

To evaluate the Segmentation Model, the pixels in an image corresponding to the medical regions of interest (ROIs) are considered true positives, while background pixels are considered true negatives. The Dice coefficient (DC) measures the similarity between the predicted results and the ground truth. High Dice scores confirm that the model's outputs closely match expert annotations, substantiating the reliability of the AI segmentation model in clinical image analysis. The DC is calculated as follows:

$$\text{Dice Coefficient} = \frac{2TP}{2TP + FP + FN}$$

9. Odds Ratio (OR)

An OR quantifies how strongly an exposure is related to a specific outcome. It compares the likelihood of the outcome happening among those who have been exposed to the factor in question with the likelihood of the same outcome occurring among those who have not been exposed.

In this study, the odds ratio measured the relative likelihood of obtaining a correct prediction (true positive or true negative) for Cutaneous Leishmaniasis, given the model's exposure to positive test samples (that is, samples from patients with confirmed Cutaneous Leishmaniasis), as opposed to when such exposure was absent (that is, when the model was tested only with samples from patients without Cutaneous Leishmaniasis).

Notably, this metric is largely unaffected by data imbalance, as it considers the proportion of correct to incorrect predictions within each group, rather than being influenced by the unequal distribution of classes. Thus, the odds ratio provides a reliable assessment of model performance, even in scenarios where class proportions are skewed.

To calculate OR, consider the following table:

		Outcome status	
		+	-
Exposure status	+	a	b
	-	c	d

a = Number of exposed cases

b = Number of exposed non-cases

c = Number of unexposed cases

d = Number of unexposed non-cases

OR is calculated as follows:

$$OR = \frac{a/c}{b/d} = \frac{ad}{bc} \qquad OR = \frac{\text{(n) exposed cases x (n) unexposed non-cases}}{\text{(n) exposed non-cases x (n) unexposed cases}}$$

References

1. Müller D, Soto-Rey I, Kramer F. Towards a guideline for evaluation metrics in medical image segmentation. BMC Res Notes. 2022 Dec;15(1):210.
2. Maxwell AE, Warner TA, Guillén LA. Accuracy Assessment in Convolutional Neural Network-Based Deep Learning Remote Sensing Studies—Part 1: Literature Review. Remote Sens. 2021 Jun 23;13(13):2450.
3. Szumilas M. Explaining Odds Ratios. J Can Acad Child Adolesc Psychiatry. 2010;19(3):227-9.
